# Common Oncogene Mutations and Novel *SND1-BRAF* Transcript Fusion in Lung Adenocarcinoma from Never Smokers

**DOI:** 10.1038/srep09755

**Published:** 2015-05-18

**Authors:** Jin Sung Jang, Adam Lee, Jun Li, Hema Liyanage, Yanan Yang, Lixia Guo, Yan W. Asmann, Peter W. Li, Michele Erickson-Johnson, Yuta Sakai, ZhiFu Sun, Hyo-Sung Jeon, Hayoung Hwang, Aaron O. Bungum, Eric S. Edell, Vernadette A. Simon, Karla J. Kopp, Bruce Eckloff, Andre M. Oliveira, Eric Wieben, Marie Christine Aubry, Eunhee Yi, Dennis Wigle, Robert B. Diasio, Ping Yang, Jin Jen

**Affiliations:** 1 Departments of Medicine, Division of Pulmonary and Critical Care Medicine, Mayo Clinic, Rochester, MN; 2 Molecular Pharmacology and Experimental Therapeutics, Mayo Clinic, Rochester, MN; 3 Department of Oncology and Hematology, China-Japan Union Hospital of Jilin University, Jilin, China; 4 Sequenom, Inc., San Diego, CA, USA; 5 Health Science Research, Mayo Clinic, Rochester, MN; 6 Laboratory Medicine and Pathology, Mayo Clinic, Rochester, MN; 7 Molecular Diagnostics and Imaging Center, School of Medicine, Kyungpook National University, Daegu, Korea; 8 New Drug Development Center, Daegu-Gyeongbuk Medical Innovation Foundation, Daegu, Korea; 9 Gene Expression Core, Mayo Clinic, Rochester, MN; 10 Biospecimen Accessioning and Processing Core, Mayo Clinic, Rochester, MN; 11 DNA Sequencing Core, Mayo Clinic, Rochester, MN; 12 Thoracic Surgery, Mayo Clinic, Rochester, MN

## Abstract

Lung adenocarcinomas from never smokers account for approximately 15 to 20% of all lung cancers and these tumors often carry genetic alterations that are responsive to targeted therapy. Here we examined mutation status in 10 oncogenes among 89 lung adenocarcinomas from never smokers. We also screened for oncogene fusion transcripts in 20 of the 89 tumors by RNA-Seq. In total, 62 tumors had mutations in at least one of the 10 oncogenes, including *EGFR* (49 cases, 55%), K-*ras* (5 cases, 6%), *BRAF* (4 cases, 5%), *PIK3CA* (3 cases, 3%), and *ERBB2* (4 cases, 5%). In addition to ALK fusions identified by IHC/FISH in four cases, two previously known fusions involving *EZR- ROS1* and *KIF5B-RET* were identified by RNA-Seq as well as a third novel fusion transcript that was formed between exons 1–9 of *SND1* and exons 2 to 3′ end of *BRAF*. This in-frame fusion was observed in 3/89 tested tumors and 2/64 additional never smoker lung adenocarcinoma samples. Ectopic expression of SND1-BRAF in H1299 cells increased phosphorylation levels of MEK/ERK, cell proliferation, and spheroid formation compared to parental mock-transfected control. Jointly, our results suggest a potential role of the novel BRAF fusion in lung cancer development and therapy.

Lung adenocarcinoma is the most common type of lung cancer worldwide and occurs in both smokers and never smokers (NS). It is estimated that up to 70% of all lung cancers in women living in East Asia are unrelated to smoking, while in North America about 15% of all lung cancers in both men and women occur in NS^1^,^2^,^3^. The different mutation spectra of lung cancer from smokers and nonsmokers have been well described and account for significantly different therapeutic responses in these patients^4^,^5^,^6^. For example, NSCLC adenocarcinomas in female never smokers tend to have a much higher frequency of EGFR gene mutations and *EML4-ALK* transcript fusions^7^. These patients benefit from targeted drugs such as gefinitib and crizotinib with significant tumor response and improved progression-free survival in advanced NSCLC over conventional therapy^7^,^8^. In contrast, up to 50% of lung adenocarcinomas from smokers carry K-*ras* gene mutations, and they often respond poorly to tyrosine kinase inhibitor (TKI) treatment^9^,^10^.

As a result of these discoveries, treatment strategies for lung adenocarcinoma in advanced stages have evolved significantly from the traditional platinum-based chemotherapy to a gene-based targeted approach for first line therapy when tumors carried targetable mutations^7^. Screening for oncogene mutations in primary lung tumors is becoming a clinical standard to guide individual treatment options and to identify new targets^11^. However, most patients treated with targeted kinase inhibitors eventually relapse, making it essential that new targets be identified in order to improve overall survival of the patients.

In this study, we used a mass spectrometry-based multiplex assay (MassArray technology, Sequenom, San Diego, CA) capable of quantitatively identifying single nucleotide changes in the DNA to screen for 187 mutations in 10 oncogenes (*EGFR, K-RAS, BRAF, ERBB2, MET*, *PIK3CA*, *AKT1/AKT2, KIT*, and *JAK2*)^12^. We then employed whole transcriptome sequencing (RNA-Seq) to screen for transcript fusions involving an oncogene. This study reports the high frequency of oncogene mutations and the identification of a novel *SND1-BRAF* fusion transcript in about 3% of lung adenocarcinoma from never smokers.

## Results

### Oncogene mutation detection in cancer tissues

We used a custom designed panel of 16 multiplex Mass Array assays to focus on oncogenes commonly mutated in lung adenocarcinoma (Supplementary Table S1). In total, 62 of the 89 tested tumors had mutations in at least one of the tested genes. *EGFR* gene mutations were detected in 49/89 cases (55%), while K-*ras* gene mutations were observed only in 5/89 cases (6%). Mutations involving *BRAF* were observed in 4 cases (5%), *PIK3CA* in 3 cases (3%), and *ERBB2* in 4 cases (5%) (Table 1 and Supplementary Table S2). Two tumors had compound mutations in the *EGFR* gene: Lu-246 with S768I and 773_V774insNPH mutations while sample Lu-243 had S768I and L858R mutations. None of the tumors with K-*ras, BRAF*, or *ERBB2* point mutations overlapped with each other or with those having *EGFR* mutations. However, all three cases with *PIK3CA* mutations also carried a mutation in the *EGFR* gene. No statistically significant correlations were observed between mutation status and the patients' age, gender, or tumor stage.

### Transcript fusion identification and validation

**Figure 1:**
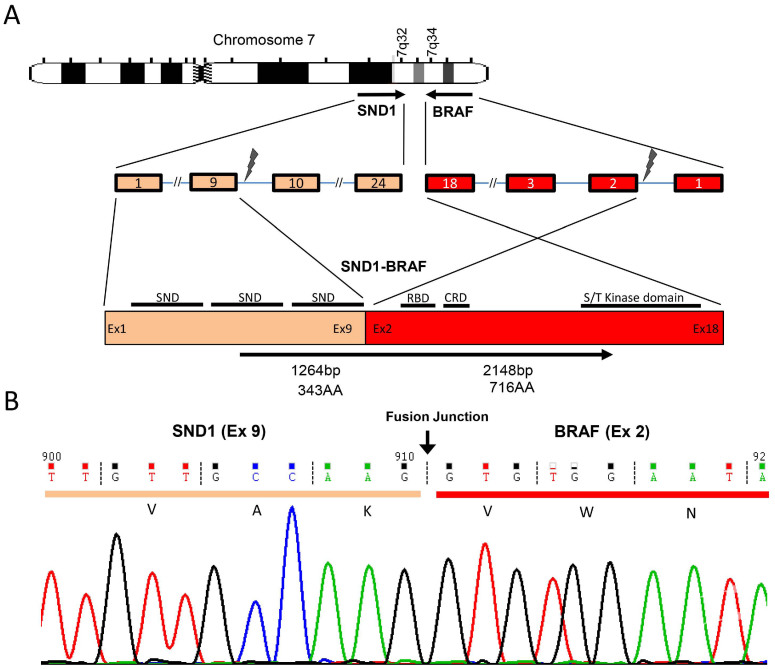
*SND1-BRAF* transcript fusion in a never smoker lung adenocarcinoma. (A). Chromosomal rearrangement at 7q32 and 7q34 results in exons 1–9 of *SND1* fused to the exon 2 to 3′ end of an inverted *BRAF*. SND, Staphylococcal nuclease domain; Ex, exon; RBD, Ras-binding domain; CRD, cysteine-rich domain; S/T, serine-threonine. (B). Sequencing traces of the RT-PCR product at fusion junction spanning exon 9 of *SND1* (orange) and exon 2 of *BRAF* (red). The dashed lines show the the reading frame.

**Figure 2:**
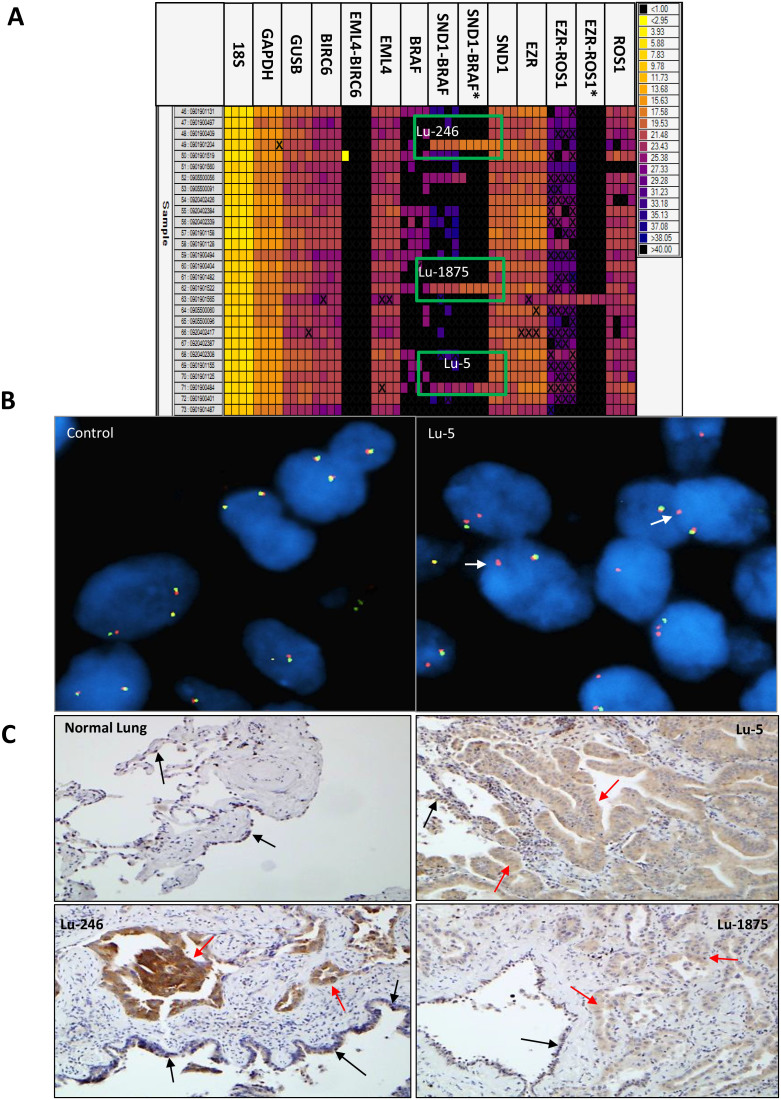
Validation and identification of additional *SND1-BRAF* fusions in lung adenocarcinomas. (A). *SND1-BRAF* fusion genes in 3 of 89 samples by qRT-PCR using Fluidigm Dynamic Array. Samples are loaded on each row and the customized assays from ABI or IDT (*) are loaded in each column as quadruplets. The targets amplified are shown at the top and the positives are indicated by boxes. (B). FISH split-apart probe flanking *BRAF* in a control and Lu-5 samples. Arrows indicate the break-apart probe adjacent to the *BRAF* gene. Magnification ×1,000. (C). IHC using anti-BRAF^wt^ antibody in normal lung and indicated tumor samples. Overexpression of BRAF in tumor cells (red arrows) was observed when comparing staining intensity with those in the adjacent bronchial epithelium cells having a basal expression level of the protein (black arrows). Magnification ×200.

**Figure 3:**
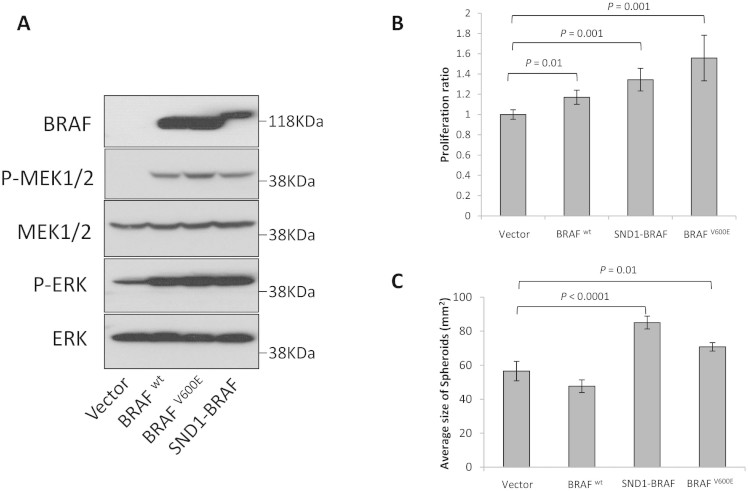
Functional Analyses of SND1-BRAF. (A). Western blots of lysates from H1299 cells transfected with pCMV6-AC-GFP vector, pCMV6-AC-GFP-BRAF^wt^, pCMV6-AC-GFP-BRAF^V600E^, or pCMV6-AC-GFP-SND1-BRAF were probed with antibodies to C-terminus of BRAF, phospho-MEK1/2 (pMEK), total MEK (MEK), phospho-ERK1/2(pERK), or total ERK (ERK). (B). Proliferation assay of H1299 cells transfected as in A. Error bars represent the average ratio from triplicates. (C). Spheroid formation assay of H1299 cell transfected with indicated constructs. Error bars represent the standard error of the mean (SEM). Statistical significances are as indicated.

Among the 20 tumors analyzed by RNA-Seq, 74 unique fusion transcripts were called and all 20 tumors had at least one fusion (Supplementary Table S3). Of those, 2 of 13 tumors without an oncogene mutation by the Mass Array assay, samples Lu-1566 and Lu-1995, had transcript fusions involving *EZR-ROS1* and *KIF5B-RET*, respectively (Table 2 and Supplementary Fig. S1). Among the 7 of 20 tumors having at least one oncogene mutation, a novel fusion transcript involving *SND1-BRAF* gene was identified in Lu-5 (Table 2 and Fig. 1). Quantitative PCR by the Fluidigm Dynamic Array confirmed the fusion in sample Lu-5 and also identified two additional tumors, Lu-246 and Lu-1875, carrying the same *SND1-BRAF* fusion (Fig. 2 and Table 3). FISH confirmed all three fusions identified by RNA-Seq involving *ROS1, RET* and *BRAF* (Supplementary Fig. S1 and Fig. 3B).

### Genomic structure of the SND1-BRAF transcript fusion in lung cancer

Sequencing analysis of the *SND1-BRAF* fusion transcript in sample Lu-5 revealed an intra-chromosomal rearrangement between the exons 1–9 of *SND1* and the exons 2-3′ end of the inverted *BRAF* on chromosome 7 (Fig. 1). The resulting fusion protein is predicted to include 1- 343AA of *SND1* and 50 - 766AA of *BRAF*. Interestingly, all three tumors with *SND1-BRAF* fusion also carried at least one other oncogene mutation. Lu-5 had an additional M774_A775insAYVM mutation in the *ERBB2* gene; Lu-246 had a mutation in the *EGFR* gene (S768I and H773_V774insNPH); while Lu-1875 had compound mutations in both *EGFR* (S768I) and *PIK3CA* (E542K) genes.

### IHC in tumor samples with SND1-BRAF fusion protein expression

We stained whole tissue sections using an antibody to the wild type of the BRAF protein by immunohistochemistry. Because *SND1* is fused to exon 2 of *BRAF*, we expected IHC to be positive using an antibody against the wild type of BRAF if the fusion transcript generated a protein product. All three tumors with the *SND1-BRAF* fusion showed an increased expression of BRAF in cancer cells compared to the adjacent non-involving bronchial epithelium present in the same section which had a basal level staining of the protein (Fig. 2C).

### DNA rearrangements involving *SND1-BRAF* fusion

We assessed the genetic rearrangement that led to the *SND1-BRAF* fusion by whole genome sequencing using a shot-gun approach (Complete Genomics, Inc, Mountain View, CA) using sample Lu-5. At 40x read depth coverage, whole genome analysis revealed multiple genomic rearrangements, consistent with chromothripsis^13^, at the region surrounding *SND1* and *BRAF* genes on chromosome 7 involving several different junction points of both genes and a third *TBXAS1* gene located between the two fusion genes (Supplementary Fig. S2). Therefore, it seems that while many DNA level changes were observed by the whole genome shotgun sequencing of the same tumor, only one productive fusion transcript was formed between *SND1-BRAF* as a result of the genomic rearrangement in the region.

Using a whole gene capture strategy for formalin-fixed, paraffin-embedded (FFPE) DNA samples, we identified two additional never smoker lung cancer cases from the same cohort also carrying the *SND1-BRAF* fusion (Table 3, Y59 and Y69). We then confirmed the presence of the same fusion in these samples by direct sequencing of the cDNA product using RNA isolated from the matched FFPE tumor samples (Table 3 and Supplementary Fig. S3).

### *SND1-BRAF* transcript increases MEK/ERK phosphorylation and oncogenic functions

In order to investigate the role of SND1-BRAF fusion protein, we examined the Raf signaling pathway by transiently expressing the fusion gene product and analyzed the phosphorylation status of the downstream proteins by western blot in the H1299 lung cancer cell line. Fig. 3A shows that the SND1-BRAF fusion protein significantly increased phosphorylation of its downstream effectors, MEK and ERK, compared to the vector control. In addition, the phosphorylation level of ERK in the SND1-BRAF fusion protein was increased similarly to BRAF^WT^ and BRAF^V600E^, an activating mutation most commonly observed in melanomas (Fig. 3A). We also observed that the SND1-BRAF fusion protein increased cell proliferation and spheroid formation compared to the vector control or BRAF^WT^ (Fig. 3B and 3C). The fusion protein demonstrated the highest spheroid formation activity in this assay, followed by BRAF^V600E^ and BRAF^WT^.

## Discussion

In this study, we used a customized Mass Array assay panel to determine the presence of oncogene mutations that are frequently observed and potentially targetable in lung adenocarcinoma from never smokers. We recently demonstrated the robustness of this customized Mass Array assay for detecting oncogene mutations in FFPE tumor samples^12^. In the 89 tumors examined in this study, 62 had a mutation in at least one of the tested genes. Furthermore, our study showed that mutations in the *EGFR* gene were the most frequent and occurred in 55% of all tested tumors (49 of 89 cases) from a population of European descent. Mutations in *KRAS, BRAF*, and *ERBB2* were much less frequent, non-overlapping, and present in tumors without with *EGFR* mutations. These results are consistent with previous findings and further supporting the dependence on EGFR signaling in lung adenocarcinoma from never smokers^8^,^14^,^15^.

In addition to identifying two previously reported fusions involving *EZR-ROS1* and *KIF5B-RET*, the unique finding of our study is the identification of a novel, in-frame fusion involving exons 1–9 of *SND1* and exons 2-3′ end of *BRAF* gene. Through a combined analysis that began with RNA-Seq and followed by validation through RT-PCR and Sanger sequencing, we additionally utilized methods such as whole genome shot-gun sequencing, whole gene-targeted capture of the entire genomic region of both genes, as well as FISH and IHC (when possible) to confirm and to identify additional cases with this new fusion. In total, we observed the *SND1-BRAF* fusion in five lung adenocarcinomas from never smoker patients. Each fusion was validated by at least two independent methods using different sample preps (Summary in Table 3).

*BRAF* mutations such as BRAF^V600E^ and its fusion transcripts lead to constitutive activation of its Ser/Thr kinase activity and down-stream activation of RAF/MEK/ERK pathway in lung, melanoma, thyroid, and colon cancers^7^,^16^,^17^,^18^,^19^. Recently, Hutchinson, et al.^18^ utilized a similarly integrative approach to identify new fusions involving *BRAF* in melanomas potentially sensitive to MEK inhibition by Trametinib using ectopically expressed *BRAF* fusion gene in the embryonic kidney HEK293 cells. In all reported *BRAF* fusions to date, the 3′ portion of the gene from exon 9 or later was involved^16^,^17^,^18^,^19^,^20^ and excluded the RAS binding domain (RBD) known to play a role in BRAF dimerization associated with the wild-type BRAF. In contrast, the *SND1-BRAF* fusion we identified in this study includes all but the first exon of BRAF. Therefore, our unique fusion is expected to retain all functional domains as the wild-type BRAF and whether it is targetable in cancer remains to be determined.

*SND1* is a component of the RNA-induced silencing (RISC) complex and plays a role as a regulator for transcription of specific mRNAs through mediating RNA interference^21^. Overexpression of *SND1* is associated with colon and prostate cancer and hepatocellular carcinoma progression^22^,^23^. Recently, Lee et al.^20^ reported observing a 979 AA *SND1-BRAF* fusion transcript between exons 1–16 of *SND1* providing the promoter and exons 9–18 of *BRAF* in a gastric cancer cell line after treatment with MET inhibitor and provides the driver of the overexpressed *BRAF* in a fusion. They further demonstrated that the *SND1-BRAF* transcript increased phosphorylation of ERK that was susceptible to MEK inhibition^20^. Similarly, our functional analysis using the 1,059 AA fusion protein demonstrated constitutive BRAF activity and down-stream activation of MEK and ERK (Fig. 3A). The preservation of all but the first exon of *BRAF* in the SND1-BRAF fusion protein identified in this study suggests a possible biological activity similar to the wild-type BRAF. Thus, RAF and MEK inhibitors such as Vemurafenib and Trametinib needs to be evaluated for their effectiveness in tumors having the *SND1-BRAF* fusion^7^,^18^,^19^,^20^.

In summary, several major findings from our study are worth noting. 1) The frequency of a targetable oncogene mutation in the *EGFR* gene is similarly high in our cohort of NS patients of European descent compared to Asian populations^1^,^2^,^3^. 2) We identified a novel transcript fusion involving exons 1–9 of *SND1* and exons 2 to 3′ end of the *BRAF* gene. 3) The fusions transcript was detected at ~3%, in five cases of the 153 (89 + 64) cases, a rate similar to that of fusions involving *ALK*, *ROS1*, and *RET* in lung adenocarcinoma. 4) Based on read evidence that span the fusion junction by RNA-Seq in Lu-5 (Table 2) and the relative peak heights by direct sequencing in Y59 and Y69 (Figure S3), the fusion transcript appeared to be present in a relatively small population of tumor cells compared to the > 60% tumor percentage in the primary tissues. 5) Four of the five tumors having the *SND1-BRAF* fusion also harbored mutations in another known driver genes suggesting a heterozygous nature of the fusion alleles in these samples (Table 3). 6) Whole genome sequencing in sample Lu-5 where sufficient DNA was available revealed complex genomic rearrangements at the region surrounding the fusion gene partners (Supplementary Fig. S2).

Finally, the relative fraction of the concurring mutations in genes known to be oncogenic within the same tumor and its clinical implications are worth further investigation. Biologically, the oncogene induced senesces is known to be associated with the active BRAF and may therefore permit only a small fraction of tumor cells to carry the fusion in most cases unless a secondary event occurs that enables the precursor tumor cells to progress through the RAS/RAF/ERK pathway via an overexpressed *SND1-BRAF* fusion. In this study, we identified a new *SND1*-*BRAF* fusion that appeared to be present in a subpopulation of tumor cells. Further studies will be needed to understand the biological and oncogenic implications of this novel fusion; how it contributes to tumor development and influence therapeutic outcome, particularly those based on targeted strategy.

## Methods

### Samples

Primary lung adenocarcinomas of never smokers (<100 cigarettes lifetime) of European descent were obtained from 89 patients who had surgery between January 1997 and September 2008. These patients were a subset of a cohort that had been previously described^24^ and chosen solely based on the availability of fresh frozen (FF) tissues having greater than 60% tumor cell purity (Table 1). Four cases were ALK positive based on immunohistochemistry (IHC) and fluorescent *in situ* hybridization (FISH)^24^. Genomic DNA was extracted with the QIAamp DNA Mini Kit (Qiagen Inc, Valencia, USA) by the Biospecimen Accessioning and Processing (BAP) Core at Mayo Clinic. Total RNA was extracted using the miRNAeasy Kit. The study was approved by the Institutional Review Board of Mayo Clinic, and signed informed consent was obtained from all participants or from patients' representatives if direct consent could not be obtained. All experiments were performed in accordance with relevant guidelines and regulations, and the Institutional Review Board of Mayo Clinic had approved all study protocols.

### Mutation identification by Mass Array assays

We first surveyed the performance of the commercially available OncoCarta^TM^ Panel that interrogates 19 oncogenes and 238 mutations. We then selected for genes having a detectable mutation in at least one tested sample and used the COSMIC database and literature to include other known somatic oncogene mutation hotspots potentially targetable for therapy. As a result, our customized panel included 187 mutations of which 134 were in OncoCarta^TM^ while 53 were new. The custom panel examines 10 oncogenes: *EGFR, K-RAS, BRAF, ERBB2, MET*, *PIK3CA*, *AKT1* and *AKT2*, *KIT*, and *JAK2*. All primers for PCR amplification and for single base extension (SBE) were designed using the Sequenom MassARRAY Design software and synthesized using standard purification protocols (Integrated DNA Technology, Coralville, IA). Amplicons were consolidated when possible to reduce their overlap with each other after PCR amplification. Primer sequences are available upon request. Each single extension probe mixture was individually evaluated by MassArray and optimized based on the Primer Adjustment Report from the MassArray Typer Analyzer 4.0 software. All individual calls were made by the Typer Analyzer 4.0 software provided by Sequenom based on peak height at the expected molecular weight; they were then manually reviewed as previously described^12^. A mutation was called positive when the peak height was ≥5% of that observed for the wild type allele. *MET* oncogene mutations known to be germ line changes were excluded from further analysis. All 89 samples were screened independently by the custom panel and all mutations were validated by Sanger sequencing (Applied Biosystems).

### Fusion transcript identification by RNA-Seq

We used RNA-Seq to analyze samples from 13 of 20 tumors without any mutation and 7 tumors with at least one mutation by the MassArray assay where sufficient total RNA were available. For each sample, 100 ng total RNA extracted from FF tumor samples were used to construct libraries using TruSeq^TM^ v2 RNA sample preparation protocol (Illumina, San Diego). Sequencing was performed on a HiSeq 2000 instrument with a 50 cycles pair-end read. FASTQ formatted raw files for each sample were mapped and aligned in reference to hg19. Fusion transcripts were identified as previously described using SnowShoes-FTD version 2.0^25^. A fusion was called when there were at least two reads that span the fusion junction and three reads that encompassing the two fusion transcripts^26^. Candidate fusion transcripts involving an oncogene were confirmed by RT-PCR and Direct sequencing.

### Fusion identification by RT-PCR, direct sequencing, and Fluidigm Dynamic Array

For reverse transcription, 100 ng total RNA was used to generate cDNA using oligo(dT) primers and the Superscript III First-Strand Synthesis kit following the manufacturer's protocol (Life Technologies, Carlsbad, CA). PCR was performed using cDNA diluted by 1:10 with RNAase free water and FastStart Taq DNA polymerase (Roche, Basel, Switzerland). The products were separated on 2% agarose gels to detect potential fusion products, then gel-extracted and sequenced to determine the exact break point sequences for each fusion.

For Fluidigm Dynamic Array analysis, TaqMan probes for 5′ and 3′ partners of the identified fusion genes and controls (*GAPDH*, *GUSB* and *18S*) were purchased from Life Technologies. The detection probes for identified fusion transcripts, such as *SND1-BRAF*, were custom designed to span fusion junctions and were obtained from both Life Technologies (Carlsbad, CA) and Integrated DNA Technologies (Coralville, IA). The sequences for all primers and probes are listed in Supplementary Table S4. Real-time PCR was performed in quadruplets using Fluidigm dynamic array as previously described^27^. The presence of a fusion by Fluidigm was considered when RT-PCR for the expected fusion transcript was uniformly positive by both assays (Life Technology and IDT).

### Fluorescence in situ Hybridization (FISH)

Rearrangements of *ROS1* (6q22) and *RET* (10q11) were independently detected using a laboratory developed dual-color break-apart probe (BAP) strategy probe set (FROS1 and FLRET, Mayo Medical Labs, Rochester, MN). 5′ and 3′ of probes were labeled with either green or red fluorescence, respectively. For detecting *BRAF* (7q34) rearrangement, bacterial artificial chromosome (BAC) clones flanking *BRAF* were obtained from the Children's Hospital Oakland Research Institute (Oakland, CA). DNA isolation, nick translation, and hybridization were performed as described previously^28^. The 5′ *BRAF* BACs were labeled with Green dUTP (Abbott Molecular, Des Plaines, IL) and included clones RP11-767F15, RP11-73H23, and RP11-715H9. The 3′ BAC RP11-577C22 was labeled with Orange dUTP (Abbott Molecular, Des Plaines, IL). Tumor samples were considered positive if ≥10% of 200 cells showed split signals.

### Immunohistochemical staining for BRAF expression

Immunohistochemical (IHC) staining was performed using a Leica Bond III Stainer (Leica, Buffalo, IL) at the Mayo Pathology Research Core. First, tissue slides were dewaxed and retrieved for 20 minutes with Bond Dewax (Leica, Buffalo, IL) and Epitope Retrieval 2 (Leica, Buffalo, IL) reagents. Then, slides were incubated for 15 minutes with a 1:400 dilution of BRAF antibody (Clone EP152Y, recognizes 70-86AA of human BRAF, Abcam). For detection, a Polymer Refine Detection System (Leica, Buffalo, IL) was used that includes the hydrogen peroxidase block, secondary antibody polymer, DAB, and Hematoxylin. Once completed, slides were rinsed for 5 minutes in tap water, dehydrated in increasing concentrations of ethyl alcohol, and xylene-treated prior to permanent coverslipping in xylene-based media.

### Whole genome shot-gun sequencing and whole gene capture sequencing

Genomic DNA from one of the tumors, Lu-5, and its uninvolved adjacent normal lung was subjected to whole genome sequencing using the shot-gun method (Complete Genomics, Inc., Mountain View, CA). Fifteen micrograms of DNA were provided and the genome was sequenced to a 40x coverage on average. The reads were mapped and reassembled by CGI tools version 1.11.0.18 to generate SNP, CNV, and structure variants relative to NCBI Build 37.1 genome^29^. SNPs were mapped to dbSNP release 132 and Cosmic v48 known SNPs. Concordance was greater than 98.5% in all calls when compared against a total of 2.5 × 10^6^ SNPs. A custom designed whole gene capture kit (SureSelect, Agilent, Santa Clara, CA) that covered the entire genomic regions of both *SND1* and *BRAF* was used to assess gene rearrangements in the targeted regions in 64 additional never smoker lung adenocarcinoma cases from the same cohort^24^. Up to 500 ng total DNA isolated from FFPE sections were used to prepare DNA libraries followed by targeted whole gene capture and sequencing using HighSeq 2000 with a 101 paired end reads protocol. The sequence reads were aligned to hg19 using Novoalign (version 2.08.01, http://www.novocraft.com/main/index.php), and realignment was subsequently carried out by GATK^30^. The structural variants of translocations were detected using CREST (v1.0) as described previously^31^.

### Plasmid, cell culture, and transfection

pCMV6-AC-GFP plasmid containing cDNA of a wild type *BRAF* and *SND1-BRAF* fusion gene were synthesized and purchased from Origene (Rockville, MD, USA). The *BRAF^V600E^* construct was generated using the QuikChange II XL Site-Directed Mutagenesis Kit (Agilent, CA, USA) and verified by Sanger sequencing. The wild type *BRAF* construct was used as a *BRAF^V600E^* mutagenesis template. The NCI-H1299 large cell lung cancer cell line with wild type of *EGFR* from a male Caucasian was purchased from ATCC (USA). H1299 cells were maintained in RPMI1640 (Life Technologies, USA) supplemented with 10% FBS (Life Technologies) and 1% antibiotics (Life Technologies). Transfection was performed by using Lipofectamine 2000 (Life Technologies) as instructed by the manufacturer.

### Western blotting

Western blotting was performed as described^32^. Briefly, cells were lysed in 10 mM Tris (pH 7.4) containing 1 mM EDTA, 0.5 mM EGTA, 150 mM NaCl, 1% Triton X-100, 50 mM NaF, 10 mM Na_4_P_2_O_7_, 10 H_2_O, 1 mM PMSF, and protease inhibitors (Santa Cruz). Lysates were quantified with BCA (Pierce). Ten micrograms of protein were separated with SDS-PAGE gel and transferred onto nitrocellulose membranes (Amersham,Hybond ECL). The membranes were blocked with 5% skim milk in TBS-Tween-20 and incubated with the diluted at 1:3000 anti-BRAF (#ab33899, Abcam), anti-MEK (#4694, Cell Signaling), anti-pMEK(#2338, Cell Signaling), anti-ERK (#4370, Cell Signaling), or anti-pERK(#4370, Cell Signaling) antibodies overnight at 4°C with gentle shaking. Protein bands were visualized with Pierce ECL substrates (Pierce).

### Cell proliferation and spheroid formation assays

Transfected H1299 cells were trypsinized and seeded in a 96-well plate at 5,000 cells/well. After 3 days in culture, cell proliferation was assayed using CellTiter 96 Aqueous Non-Radioactive Cell Proliferation Assay (Promega, USA) with measurement absorbance at 490 nm (Tecan, Switzerland). For spheroid formation assay^33^, transfected cells were prepared as single cells by trypsinization and cell number was adjusted at 2.5 × 10^4^ cells/ml. Twenty microliters of single cell suspensions was dropped on the lid of a 100 mm culture dish (500 cells/drop), and the drops were placed sufficiently apart so as not to touch. The lid was then inverted onto the PBS-filled bottom dish and cultured for 5 days. The size of spheroids was measured under a microscope (Zeiss, Germany).

## Additional Information

**How to cite this article**: Jang, J.S. *et al*. Common Oncogene Mutations and Novel SND1-BRAF Transcript Fusion in Lung Adenocarcinoma from Never Smokers. *Sci. Rep.* **5**, 9755; doi: 10.1038/srep09755(2015).

**Funding:** Supported by funding from the American Cancer Society, the National Foundation for Cancer Research Hillsberg Lung Cancer Translational Research Grant, the Mayo Clinic Cancer Center,the Mayo Center for Individualized medicine, and start-up founds to Dr. Yanan Yang from the Mayo Clinic Foundation.

## Supplementary Material

Supplementary Information Supplemental Data

## Figures and Tables

**Table 1:** Summary of clinical information and mutation status for 89 tested samples

Variable*		No. of Patients	*EGFR* (%)	*BRAF*^2^ (%)	*ERBB2* (%)	K-*ras* (%)	*PIK3CA* (%)	Other Fusions^3^ (%)
		(Adenocarcinoma^1^)						
Total		89	49 (55.0)	7 (7.9)	4 (4.5)	5 (5.6)	3 (3.4)	6 (6.7)
Sex								
	Male	15	5 (33.3)	3 (20.0)	0 (0)	1 (6.7)	0 (0)	4 (26.7)
	Female	74	44 (59.5)	4 (5.4)	4 (5.4)	4 (5.4)	3 (4.1)	2 (2.7)
Age								
	≥65	56	32 (57.1)	5 (8.9)	2 (3.6)	4 (12.5)	3 (5.4)	2 (3.6)
	<65	33	17 (51.5)	2 (6.1)	2 (6.1)	1 (3.0)	0 (0)	4 (12.1)
Staging								
	I	57	33 (57.9)	6 (10.5)	4 (7.0)	4 (7.0)	1 (1.8)	2 (3.5)
	II	9	4 (44.4)	0 (0)	0 (0)	0 (0)	1 (11.1)	0 (0)
	III	19	10 (52.6)	1 (5.3)	0 (0)	0 (0)	1 (5.3)	4 (21.1)
	IV	4	2 (50.0)	0 (0)	0 (0)	1 (25.0)	0 (0)	0 (0)

1Adenocarcinoma, Adeno with bronchioloalveolar carcinoma, Bronchioloalveolar carcinoma and Adenosquamous carcinoma.

2Includes *BRAF* point mutations and *SND1-BRAF* transcript fusions.

3Includes ALK+, *EZR-ROS1* and *KIF5B-RET*.

*No statistically significant difference in mutation distributions for the clinical variables shown.

**Table 2:** Fusion transcripts identified by RNA-Seq that involved known or potential targetable genes

Sample	Fusion gene directional	Type	Fusion Strand	Encompassing Read Pairs	Total SplitReads	Exon Boundary Fusion	5′ gene Information	3′ Gene Information
Lu-5	*SND1->BRAF*	intra-chr	+	29	4	YES	E9:chr7:*SND1*	E2:chr7:*BRAF*
Lu-1566	*EZR->ROS1*	intra-chr	−	108	153	YES	E10:chr6:*EZR*	E34:chr6:*ROS1*
Lu-1995	*KIF5B->RET*	intra-chr	−	144	82	YES	E15:chr10:*KIF5B*	E12:chr10:*RET*

**Table 3:** Lung adenocarcinomas having *SND1-BRAF* fusion genes

Sample ID	Sample Type	Methods of Detection	Supporting Reads#	Sanger Sequencing*	5' Gene	Break Point	3' Gene	Break Point	Other Mutations
Lu-5	FF, RNA/DNA	RNA-Seq, qRT-PCR, WGSeq, FISH	29	+	Ex9, SND1	Chr.7, 127347701	Ex2, BRAF	Chr7. 140550012	ERBB2, M774_A775insAYVM
Lu-1875	FF, RNA	qRT-PCR, IHC	−	+	Ex9, SND1	Chr.7, 127347701	Ex2, BRAF	Chr7. 140550012	EGFR, S768I, PIK3CA, E542K
Lu-246	FF, RNA	qRT-PCR, IHC	−	+	Ex9, SND1	Chr.7, 127347701	Ex2, BRAF	Chr7. 140550012	EGFR S768I, H773_V774insNPH
Y59	FFPE, DNA/RNA	Gene Capture, qRT-PCR	17	+	Ex9, SND1	Chr.7, 127347703	Ex2, BRAF	Chr7. 140550015	EML4-ALK
Y69	FFPE, DNA/RNA	Gene Capture, qRT-PCR	20	+	Ex9, SND1	Chr.7, 127347704	Ex2, BRAF	Chr7. 140550014	N.F.

#NextGen sequencing reads that either span the fusion junction of the transcript in Lu-5 or mapped to the corresponding genomic regions in Y59 and Y69.

*Sanger sequencing was performed using cDNA. N.F., Not Found.
