# Supplementary material for: Common Oncogene Mutations and Novel SND1-BRAF Transcript Fusion in Lung Adenocarcinoma from Never Smokers
Source: Sci Rep. 2015 May 18;5:9755. doi: 10.1038/srep09755 (PMC4434945; doi:10.1038/srep09755)
Supplement: Supplementary Information — Supplemental Data [file srep09755-s1.pdf]

Common Oncogene Mutations and Novel *SND1-BRAF* Transcript Fusion in Lung Adenocarcinoma from Never Smokers

*Jin Sung Jang[1], *Adam Lee[2], Jun Li[1,3], Hema Liyanage[4], Yanan Yang[1], Lixia Guo[1], Yan W. Asmann[5], Peter W. Li[5], Michele Erickson-Johnson[6], Yuta Sakai[6], ZhiFu Sun[5], Hyo-Sung Jeon[7], Hayoung Hwang[8], Aaron O. Bungum[1], Eric S. Edell[1], Vernadette A. Simon[9], Karla J. Kopp[10], Bruce Eckloff[11], Andre M. Oliveira[6], Eric Wieben[11], Marie Christine Aubry[6], Eunhee Yi[6], Dennis Wigle[12], Robert B. Diasio[2], Ping Yang[5], and Jin Jen[1,6,9]

[1]Departments of Medicine, Division of Pulmonary and Critical Care Medicine, [2]Molecular Pharmacology and Experimental Therapeutics, [5]Health Science Research, [6]Laboratory Medicine and Pathology, and [12]Thoracic Surgery; [9]Gene Expression Core, [10]Biospecimen Accessioning and Processing Core, and [11]DNA Sequencing Core, Mayo Clinic, Rochester, MN, [3]Department of Oncology and Hematology, China-Japan Union Hospital of Jilin University, Jilin, China, [4]Sequenom, Inc., San Diego, CA, USA, [7]Department of Biochemistry and Cell Biology, School of Medicine, Kyungpook National University, Daegu, and [8]New Drug Development Center, Daegu-Gyeongbuk Medical Innovation Foundation, Daegu, Korea.

Correspondence to: Jin Jen, MD, PhD, Stabile Research Building, Mayo Clinic, 200 First St SW, Rochester, MN 55905 (jen.jin@mayo.edu).

Funding: Supported by funding from the American Cancer Society, the Mayo Clinic Cancer Center, and the Mayo Center for Individualized Medicine.

Competing Interests: HL is an employee of Sequenom. All other authors have no competing interests exist.

* These authors contributed equally to this work

Supplementary Table S1. Number of detectable mutations for genes examined by OncoCarta™ and Custom Mass Array Panel.

Gene	[a]OncoCarta™	Custom Mass Array
EGFR	53	63
BRAF	24	47
MET[b]	5	21
PIK3CA	14	17
K-RAS	17	15
ERBB2	8	11
AKT1	7	7
AKT2	2	2
KIT	33	3
JAK	1	1
Total	164	187

[a] Only genes shared with Custom Mass Array are shown.

[b] Included germ line changes.

Supplementary Table S2. Mutation profiling of NS lung cancer.

Sample	EGFR_Exon	EGFR_1	EGFR_2	BRAF	ERBB2	KRAS	MET[3]	PIK3CA	Fusion Gene
Lu-0005					M774_A775insAYVM				SND1-BRAF
Lu-0106	20	V769_D770insASV							
Lu-0124	19	E746_S752A							
Lu-0132	19	L747_S752del							
Lu-0161						G12V			
Lu-0243	21, 20	L858R	S768I						
Lu-0246	20	H773_V774insNPH	S768I						SND1-BRAF
Lu-0253	18	G719A[1]							
Lu-0346	19	E746_S752A							
Lu-0389	19	E746_S752A							
Lu-0426	21	L858R							
Lu-0443	21	L858R							
Lu-0465							N375S		
Lu-0516	19	E746_A750del							
Lu-0587	19	L747_S752del							
Lu-0593				V600E					
Lu-0597									ALK+
Lu-0733	21	L858R							
Lu-0757					G776VC				
Lu-0760	21	L858R							
Lu-0789									ALK+
Lu-0824	19	E746_A750del					T1010I		
Lu-0891	21	L747_S752del							ALK+
Lu-0905	21	L858R							
Lu-0920	19	E746_A750del							
Lu-1014	19	E746_S752A							
Lu-1015				G469V					
Lu-1031					G776VC				
Lu-1033	19	E746_T751del, V ins							
Lu-1068	21	L858R					R988C		
Lu-1096	21	L858R						H1047R	

Lu-1155	19	E746_A750del						
Lu-1222	21	L858R						
Lu-1271	19	E746_A751del				E168D		
Lu-1286	19	E746_A750del						
Lu-1321	19	E746_S752A						
Lu-1333	19	E746_A750del						
Lu-1377	19	H773_V774insPH						
Lu-1437	19	E746_A750del						
Lu-1450					Q61H			
Lu-1479	19	E746_T751del, V ins				T1010I		
Lu-1518	19	E746_A750del						
Lu-1547	21	L858R						
Lu-1566								EZR-ROS
Lu-1606	21	L858R						
Lu-1632	21	L858R						
Lu-1659	19	E746_A750del						
Lu-1660			V600E			T1010I		
Lu-1682					G12D			
Lu-1683	19	E746_A750del				E168D		
Lu-1686					G12A			
Lu-1789						R988C		ALK+
Lu-1790			K601E					
Lu-1806	21	L858R						
Lu-1848					G12D			
Lu-1875	20	S768I[2]					E542K	SND1-BRAF
Lu-1908	19	E746_A750del						
Lu-1939	19	E746_A750del					E545K	
Lu-1942	21	L858R						
Lu-1943	19	E746_S752A						
Lu-1995								KIF5B-RET
Lu-2029	19	E746_A750del						
Lu-2040				M774_A775insAYVM				
Lu-2070	20	T790M						

Lu-2172	19	E746_S752A						
Lu-2196	21	L858R				N375S		
Lu-2456	21	L858R						
Lu-2502	19	E746_A750del						
Total	68	49	4	4	5	9	3	9

[1] OncoCartaTM detected. [2] LuCaMSP detected. [3]Germ line changes. ALK$^+$ were described based on as previous result (12).

Supplementary Table S3. Fusion gene profiling of NS lung cancer by RNA-Seq.

Sample Name	Fusion Gene Direction	Type	Fusion Strand	Encompassing Read Pairs	Total Split Reads	Exon Boundary Fusion	5' Gene Information	3' Gene Information
Lu-0005	HIPK2->TSPAN12	intra-chr	-	51	17	YES	E1:chr7:HIPK2:NM_022740:139477403:139477693:-	E8:chr7:TSPAN12:NM_012338:120427373:120428951:-
	SND1->BRAF	intra-chr	+	29	4	YES	E9:chr7:SND1:NM_014390:127347610:127347701:+	E2:chr7:BRAF:NM_004333:140549910:140550012:-
Lu-0124	CDKL2->TBC1D1	intra-chr	-	22	13	YES	E10:chr4:CDKL2:NM_003948:76521430:76521524:-	E20:chr4:TBC1D1:NM_001253912:38134704:38134878:+
	DNAJC24->ELP4	intra-chr	+	18	9	YES	E2:chr11:DNAJC24:NM_181706:31392262:31392406:+	E4:chr11:ELP4:NM_019040:31616316:31616448:+
	FAM190A->WDFY3	intra-chr	+	6	6	YES	E1:chr4:FAM190A:NM_001145065:91048683:91048982:+	E5:chr4:WDFY3:NM_014991:85771054:85771178:-
	FLJ43663->WDR91	intra-chr	-	41	20	YES	E1:chr7:FLJ43663:NR_015431:130792478:130793562:-	E7:chr7:WDR91:NM_014149:134882744:134882903:-
	LOC100131691->OTC	inter-chr	+	20	20	YES	E1:chr19:LOC100131691:NR_027334:59070552:59070776<br>:+	E5:chrX:OTC:NM_000531:38260527:38260681:+
	LOC730102->SEC16B	intra-chr	-	5	1	YES	E7:chr1:LOC730102:NR_037167:177995289:177995390:-	E2:chr1:SEC16B:NM_033127:177936817:177937174:-
	LRRC28->TPST1	inter-chr	+	9	3	YES	E3:chr15:LRRC28:NM_144598:99816780:99816821:+	E3:chr7:TPST1:NM_003596:65751497:65751696:+
	SMARCC2->CALCOCO2	inter-chr	-	8	4	YES	E7:chr12:SMARCC2:NM_139067:56577644:56577714:-	E11:chr17:CALCOCO2:NM_005831:46937675:46937811:+
	TESC->FBXO21	intra-chr	-	8	5	YES	E1:chr12:TESC:NM_017899:117537029:117537251:-	E5:chr12:FBXO21:NM_033624:117612455:117612602:-
	ZNF516->SYTL5	inter-chr	-	6	3	YES	E2:chr18:ZNF516:NM_014643:74174983:74175097:-	E2:chrX:SYTL5:NM_138780:37892786:37893261:+
Lu-0155	NDE1->C16orf45	intra-chr	+	57	30	YES	E5:chr16:NDE1:NM_001143979:15771657:15771806:+	E2:chr16:C16orf45:NM_033201:15609161:15609285:+
	TFG->GPR128	intra-chr	+	83	25	YES	E3:chr3:TFG:NM_001007565:100438818:100438902:+	E2:chr3:GPR128:NM_032787:100348441:100348555:+
Lu-0158	LOC730102->SEC16B	intra-chr	-	6	2	YES	E7:chr1:LOC730102:NR_037167:177995289:177995390:-	E2:chr1:SEC16B:NM_033127:177936817:177937174:-
Lu-0465	CNOT2->TSPAN8	intra-chr	+	17	7	YES	E2:chr12:CNOT2:NM_014515:70671911:70672054:+	E2:chr12:TSPAN8:NM_004616:71551398:71551567:-
	FRS2->MDM1	intra-chr	+	25	5	YES	E1:chr12:FRS2:NM_006654:69864128:69864310:+	E2:chr12:MDM1:NM_017440:68724886:68725001:-
	SSPN->TSPAN8	intra-chr	+	54	16	YES	E1:chr12:SSPN:NM_001135823:26348268:26348464:+	E2:chr12:TSPAN8:NM_004616:71551398:71551567:-
Lu-0597	LOC285629->ATP10B	intra-chr	-	12	2	YES	E2:chr5:LOC285629:NR_027111:160362996:160363116:-	E2:chr5:ATP10B:NM_025153:160212565:160212810:-
Lu-0847	TSPAN9->NCAPD2	intra-chr	+	23	8	YES	E2:chr12:TSPAN9:NM_001168320:3310342:3310422:+	E18:chr12:NCAPD2:NM_014865:6634748:6634882:+
Lu-0891	ATF7IP2->NUBP1	intra-chr	+	12	8	YES	E1:chr16:ATF7IP2:NM_001256160:10479911:10479979:+	E10:chr16:NUBP1:NM_002484:10861805:10861889:+
	CDH3->TEPP	intra-chr	+	7	5	YES	E2:chr16:CDH3:NM_001793:68679538:68679653:+	E3:chr16:TEPP:NM_199046:58018206:58018268:+
	CYP4B1->CYP4X1	intra-chr	+	12	9	YES	E8:chr1:CYP4B1:NM_001099772:47280745:47280936:+	E2:chr1:CYP4X1:NM_178033:47495661:47495803:+
	EML4->BIRC6	intra-chr	+	37	9	YES	E5:chr2:EML4:NM_001145076:42491845:42491871:+	E69:chr2:BIRC6:NM_016252:32822817:32823035:+
	FBXO11->ATAD2B	intra-chr	-	14	2	YES	E4:chr2:FBXO11:NM_001190274:48065997:48066142:-	E20:chr2:ATAD2B:NM_001242338:24011381:24011517:-
Lu-0918	KRBOX1->FAM198A	intra-chr	+	6	1	YES	E4:chr3:KRBOX1:NM_001205272:42983449:42983563:+	E3:chr3:FAM198A:NM_001129908:43095012:43095135:+
	LINC00189->BACH1	intra-chr	+	52	7	YES	E2:chr21:LINC00189:NR_027072:30566436:30566581:+	E2:chr21:BACH1:NM_001186:30693541:30693835:+
	TAX1BP1->UTP6	inter-chr	+	80	47	YES	E5:chr7:TAX1BP1:NM_001206901:27809294:27809453:+	E3:chr17:UTP6:NM_018428:30221988:30222030:-
Lu-0920	NPC2->SYNDIG1L	intra-chr	-	13	1	YES	E4:chr14:NPC2:NM_006432:74947404:74947482:-	E2:chr14:SYNDIG1L:NM_001105579:74876030:74876504:-
	SHFM1->LOC100506136	intra-chr	-	6	6	YES	E2:chr7:SHFM1:NM_006304:96324109:96324203:-:-	E5:chr7:LOC100506136:NR_038948:96250968:96251955

Lu-1208	CHD9->RLTPR	intra-chr	+	18	11	YES	E19:chr16:CHD9:NM_025134:53289511:53289691:+	E33:chr16:RLTPR:NM_001013838:67689933:67689970:+
	FAM189A2->PARD6G	inter-chr	+	8	7	YES	E2:chr9:FAM189A2:NM_001127608:71951128:71951185:+	E2:chr18:PARD6G:NM_032510:77960592:77960815:-
	RASEF->HSD17B3	intra-chr	-	19	12	YES	E5:chr9:RASEF:NM_152573:85627348:85627426:-	E3:chr9:HSD17B3:NM_000197:99017149:99017225:-
	TBC1D14->STX18	intra-chr	+	20	19	YES	E2:chr4:TBC1D14:NM_001113361:6925099:6925838:+	E2:chr4:STX18:NM_016930:4473365:4473433:-
Lu-1431	CCT2->BRD9	inter-chr	+	41	27	YES	E10:chr12:CCT2:NM_006431:69987289:69987393:+	E10:chr5:BRD9:NR_027633:879908:880004:-
	MPP6->TOMM7	intra-chr	+	7	2	YES	E6:chr7:MPP6:NM_016447:24690103:24690331:+	E2:chr7:TOMM7:NM_019059:22857618:22857667:-
	NCOA2->ADAMTS2	inter-chr	-	11	2	YES	E1:chr8:NCOA2:NM_006540:71315934:71316020:-:-	E4:chr5:ADAMTS2:NM_014244:178634513:178634716
	TPPP->TRIP13	intra-chr	-	6	4	YES	E1:chr5:TPPP:NM_007030:693392:693510:-	E2:chr5:TRIP13:NM_001166260:894901:895067:+
Lu-1566	C12orf23->SLC35F1	inter-chr	+	16	2	YES	E3:chr12:C12orf23:NM_152261:107360888:107360958:+	E2:chr6:SLC35F1:NM_001029858:118475607:118475783:+
	EZR->ROS1	intra-chr	-	108	153	YES	E10:chr6:EZR:NM_001111077:159191795:159191926:-	E34:chr6:ROS1:NM_002944:117645494:117645578:-
	ROS1->HIPK2	inter-chr	-	51	4	YES	E28:chr6:ROS1:NM_002944:117663562:117663707:-	E5:chr7:HIPK2:NM_022740:139313698:139313785:-
Lu-1825	CLPTM1L->NSMCE2	inter-chr	-	17	18	YES	E2:chr5:CLPTM1L:NM_030782:1344465:1344566:-	E6:chr8:NSMCE2:NM_173685:126369460:126369561:+
	RAP1B->LYZ	intra-chr	+	6	3	YES	E2:chr12:RAP1B:NM_015646:69042478:69042561:+	E2:chr12:LYZ:NM_000239:69743887:69744052:+
Lu-1875	C14orf43->ACER3	inter-chr	-	6	8	YES	E3:chr14:C14orf43:NM_001043318:74203700:74204000:-	E2:chr11:ACER3:NM_018367:76637600:76637711:+
	CRTC3->ZNF592	intra-chr	+	9	2	YES	E1:chr15:CRTC3:NM_001042574:91073197:91073435:+	E2:chr15:ZNF592:NM_014630:85307937:85308046:+
	CYP4B1->CYP4X1	intra-chr	+	7	1	YES	E8:chr1:CYP4B1:NM_001099772:47280745:47280936:+	E2:chr1:CYP4X1:NM_178033:47495661:47495803:+
	KDM2A->PPP6R3	intra-chr	+	32	24	YES	E2:chr11:KDM2A:NM_012308:66888704:66888829:+	E5:chr11:PPP6R3:NM_018312:68312305:68312492:+
	LOC100506393->SARNP	intra-chr	+	31	18	YES	E2:chr12:LOC100506393:NR_040098:20172565:20172630:+	E10:chr12:SARNP:NR_026723:56154334:56154424:-
	PPP6R3->SYVN1	intra-chr	+	22	13	YES	E3:chr11:PPP6R3:NM_018312:68286968:68287119:+	E10:chr11:SYVN1:NM_172230:64898390:64898507:-
	TTC3->FAM69A	inter-chr	+	11	3	YES	E31:chr21:TTC3:NM_001001894:38534291:38534365:+	E2:chr1:FAM69A:NM_001252273:93341852:93341987:-
Lu-1908	LOC730102->SEC16B	intra-chr	-	5	2	YES	E7:chr1:LOC730102:NR_037167:177995289:177995390:-	E2:chr1:SEC16B:NM_033127:177936817:177937174:-
	NPC2->SYNDIG1L	intra-chr	-	11	3	YES	E4:chr14:NPC2:NM_006432:74947404:74947482:-	E2:chr14:SYNDIG1L:NM_001105579:74876030:74876504:-
	SGK196->HGSNAT	intra-chr	+	22	4	YES	E4:chr8:SGK196:NM_032237:42958670:42958973:+	E6:chr8:HGSNAT:NM_152419:43024315:43024385:+
Lu-1939	MYO9B->FZR1	intra-chr	+	5	6	YES	E35:chr19:MYO9B:NM_001130065:17317921:17318053:+	E2:chr19:FZR1:NM_016263:3522953:3523056:+
	PARP6->MYOM1	inter-chr	-	429	171	YES	E1:chr15:PARP6:NM_020214:72563363:72563628:-	E34:chr18:MYOM1:NM_003803:3079176:3079340:-
	SH2D3A->ZNF562	intra-chr	-	5	5	YES	E1:chr19:SH2D3A:NM_005490:6767397:6767523:-	E2:chr19:ZNF562:NM_017656:9771395:9771550:-
	TRIP12->AP1S3	intra-chr	-	22	17	YES	E33:chr2:TRIP12:NM_004238:230650472:230650571	E2:chr2:AP1S3:NM_001039569:224642407:224642586:-:-
Lu-1942	KPNB1->AP2B1	intra-chr	+	7	16	YES	E12:chr17:KPNB1:NM_002265:45748067:45748198:+	E17:chr17:AP2B1:NM_001282:34009713:34009843:+
	LRIG3->MSRB3	intra-chr	-	15	2	YES	E15:chr12:LRIG3:NM_153377:59271181:59271634:-	E5:chr12:MSRB3:NM_198080:65847507:65847605:+
	LRIG3->TMEM97	inter-chr	-	51	23	YES	E3:chr12:LRIG3:NM_153377:59307762:59307837:-	E2:chr17:TMEM97:NM_014573:26652528:26652673:+
	MAML2->SESN3	intra-chr	-	7	4	YES	E1:chr11:MAML2:NM_032427:96074546:96076344:-	E2:chr11:SESN3:NM_144665:94926620:94926686:-
	MGLL->SLC41A3	intra-chr	-	45	3	YES	E3:chr3:MGLL:NM_007283:127500631:127500738:-	E2:chr3:SLC41A3:NM_017836:125786789:125787089:-
	MIB1->ZNF714	inter-chr	+	8	7	YES	E4:chr18:MIB1:NM_020774:19353584:19353689:+	E3:chr19:ZNF714:NM_182515:21280990:21281117:+

	MPRIP->RPH3AL	intra-chr	+	14	4	YES	E1:chr17:MPRIP:NM_201274:16946073:16946285:+	E7:chr17:RPH3AL:NM_006987:96901:97076:-
	NUP107->PRIM2	inter-chr	+	8	7	YES	E27:chr12:NUP107:NM_020401:69135592:69135760:+	E8:chr6:PRIM2:NM_000947:57372287:57372355:+
	SHFM1->LOC100506136	intra-chr	-	10	3	YES	E2:chr7:SHFM1:NM_006304:96324109:96324203:-:-	E4:chr7:LOC100506136:NR_038948:96275035:96275248
	TPX2->BCL2L1	intra-chr	+	13	5	YES	E1:chr20:TPX2:NM_012112:30326903:30327424:+	E3:chr20:BCL2L1:NM_138578:30252260:30253889:-
	USP15->MLIP	inter-chr	+	14	5	YES	E4:chr12:USP15:NM_006313:62708570:62708697:+	E2:chr6:MLIP:NM_138569:53986244:53986400:+
	ZMIZ1->KIAA1274	intra-chr	+	5	9	YES	E4:chr10:ZMIZ1:NM_020338:80921809:80921890:+	E2:chr10:KIAA1274:NM_014431:72285678:72285892:+
Lu-1995	EFCAB2->SMYD3	intra-chr	+	35	17	YES	E1:chr1:EFCAB2:NR_026586:245133283:245133745:+	E9:chr1:SMYD3:NM_022743:246027100:246027188:-
	EPSTI1->ENOX1	intra-chr	-	52	13	YES	E8:chr13:EPSTI1:NM_033255:43491676:43491760:-	E15:chr13:ENOX1:NM_017993:43810771:43810874:-
	KIF5B->RET	intra-chr	-	144	82	YES	E15:chr10:KIF5B:NM_004521:32317355:32317499:-	E12:chr10:RET:NM_020975:43612031:43612179:+
	KRBOX1->FAM198A	intra-chr	+	9	1	YES	E4:chr3:KRBOX1:NM_001205272:42983449:42983563:+	E2:chr3:FAM198A:NM_001129908:43073758:43075045:+
	LOC285629->ATP10B	intra-chr	-	9	3	YES	E2:chr5:LOC285629:NR_027111:160362996:160363116:-	E2:chr5:ATP10B:NM_025153:160212565:160212810:-
	MPRIP->SPNS3	intra-chr	+	201	55	YES	E3:chr17:MPRIP:NM_201274:16981324:16981390:+	E9:chr17:SPNS3:NM_182538:4381866:4381932:+
	NCOR1->DRG2	intra-chr	-	21	4	YES	E1:chr17:NCOR1:NM_006311:16118675:16118874:-	E3:chr17:DRG2:NM_001388:18001583:18001673:+
Lu-2045	ACSS3->MGAT4C	intra-chr	+	16	5	YES	E8:chr12:ACSS3:NM_024560:81568566:81568718:+	E3:chr12:MGAT4C:NM_013244:86727842:86727905:-
	CTF1->CPPED1	intra-chr	+	6	3	YES	E2:chr16:CTF1:NM_001330:30910735:30910854	E2:chr16:CPPED1:NM_001099455:12875041:12875260:-:+
	GMEB2->TERT	inter-chr	-	10	3	YES	E2:chr20:GMEB2:NM_012384:62250619:62250807:-	E4:chr5:TERT:NM_198253:1280272:1280453:-
	KRBOX1->FAM198A	intra-chr	+	5	1	YES	E4:chr3:KRBOX1:NM_001205272:42983449:42983563:+	E3:chr3:FAM198A:NM_001129908:43095012:43095135:+
	MEF2D->GOLPH3L	intra-chr	-	21	7	YES	E1:chr1:MEF2D:NM_005920:156470277:156470529	E3:chr1:GOLPH3L:NM_018178:150636107:150636239:-:-
	TPPP->BRD9	intra-chr	-	8	12	YES	E1:chr5:TPPP:NM_007030:693392:693510:-	E10:chr5:BRD9:NR_027633:879908:880004:-

Supplementary Table S4. Sequences of PCR primers and probes for fusion transcripts detection.

Fusion gene	Forward	Reverse	TaqMan Probe
EZR-ROS1	AGCTCAGCCAACTCTTTGTCT	AGAAAAGGAGAGAAACCGTGGA	
EML4-BIRC6	CATCACCAGCTGAAAAGTCACA	GAGGATGAAGACAGAGGCAGT	
KIF5B-RET	GCGAGTATAGATGCTGAGCTTC	TGCTCTGCCTTTCAGATGGAA	
SND1-BRAF	GTGTGGACTGGTCGATTGCA	GGTGTATTCTTCATAGGCCTCCA	
SND1-BRAF	GCAGGCTGAGAATATGGAGAG	GGCCTCTATATGTTCCTGTGTC	ACAAGCAGTTTGTTGCCAAGGTGTGG

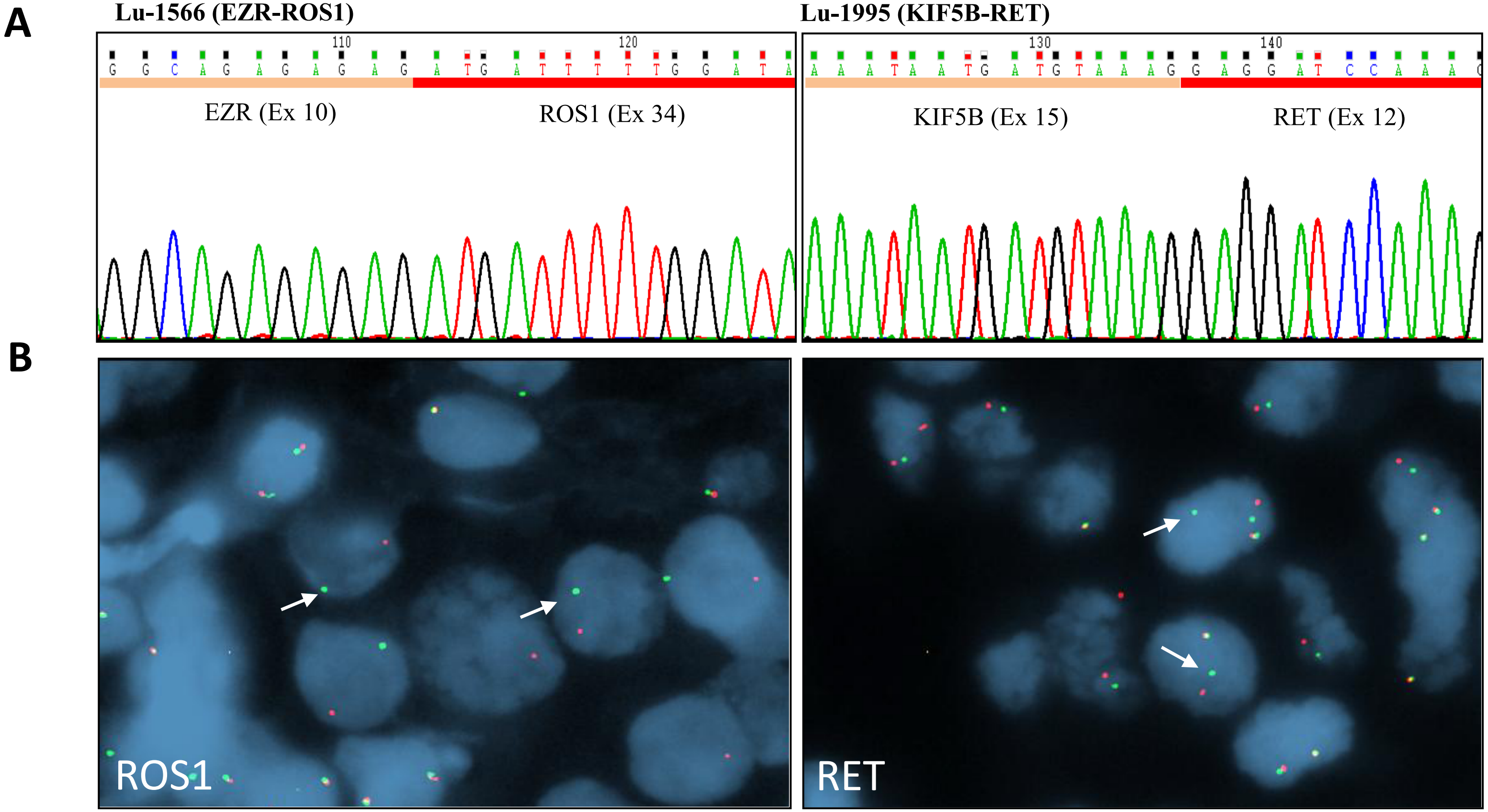

Supplementary Figure S1. Confirmation of fusion transcripts. A. Sanger sequencing traces of fusions involving *EZR* (orange)-*ROS1*(red) and *KIF5B*(orange)-*RET*(red) genes in the samples indicated. B. FISH validation of fusions using break-apart probes. Arrows indicate the break-apart probe adjacent to the *ROS* or *RET* genes, respectively. Magnification x1000.

Chromosome	5' Gene	Position	Location	Strand	3' Gene	Position	Location	Strand	Type	Genomic Fusion Gene
**chr7**	**SND1**	**127361983**	**Intron10**	**-**	**BRAF**	**140572145**	**Intron 01**	**-**	**fusion**	**SND1-BRAF**
**chr7**	SND1	127453469	Intron11	-	TBXAS1	139517148	Intron 04	-	fusion	SND1-TBXAS1
**chr7**	SND1	127457072	Intron11	-	TBXAS1	139511559	Intron 04	-	fusion	SND1-TBXAS1
**chr7**	SND1	127457102	Intron11	-	BRAF	140572118	Intron 01	-	fusion	SND1-BRAF
**chr7**	TBXAS1	139705930	Intron09	+	BRAF	140582144	Intron 01	+	fusion	TBXAS1-BRAF

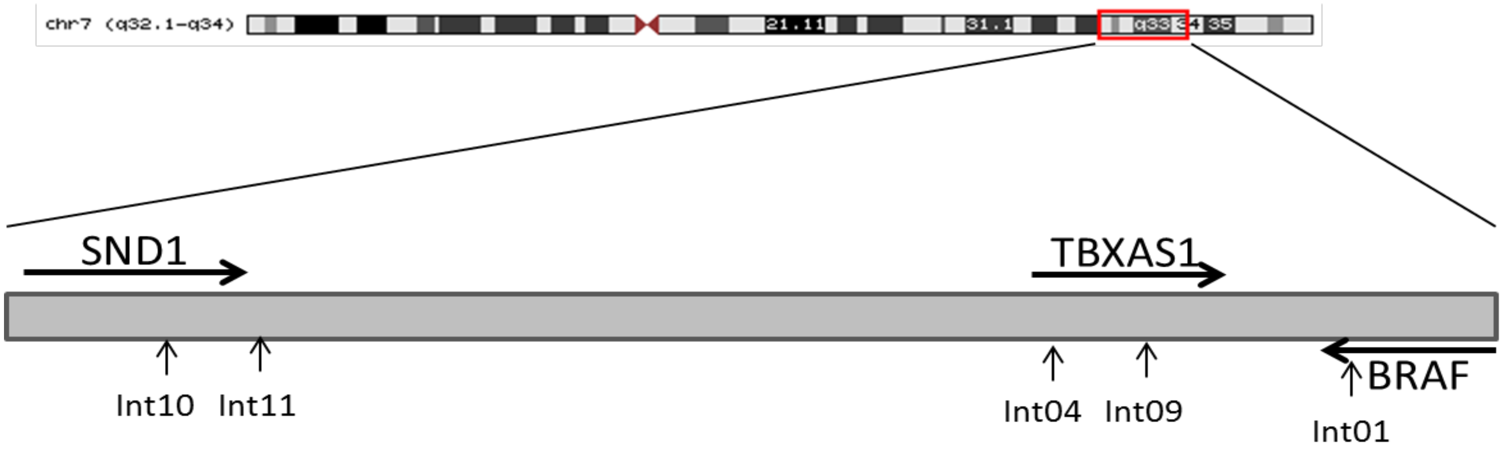

Supplementary Figure S2. Genomic rearrangements of the fusion region in Lu-5 based on whole genome sequencing. Int = intron.

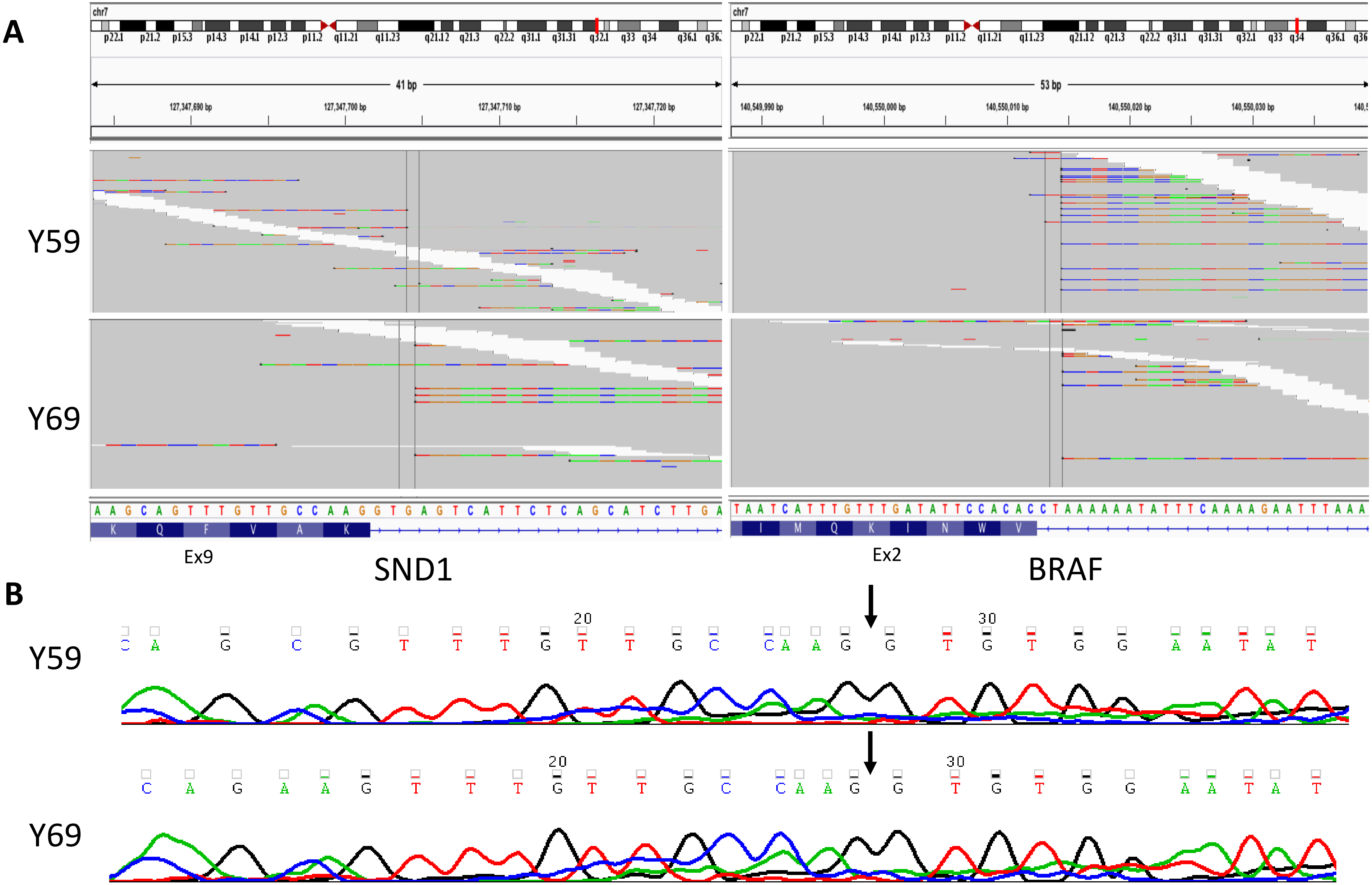

Supplementary Figure S3. A, The IGV screen shot of genomic rearrangements for the fusion region in Y59 and Y69 based on whole genome capture sequencing. The vertical lines indicated the break points. Perfectly matched reads are shown in gray. Colors represent mismatched bases soft-clipped around the break points. Both samples were broken and fused at intron 9 of SND1 and intron 1 of BRAF. **B**, Sanger sequencing using cDNA from Y59 and Y69. *SND1-BRAF* fusion transcript was confirmed by qRT-PCR and sequenced at the fusion junction spanning exon 9 of *SND1* and exon 2 of *BRAF.* Arrow heads, fusion point.
